# Mapping polyclonal antibody responses to bacterial infection using next generation phage display

**DOI:** 10.1038/srep24232

**Published:** 2016-04-13

**Authors:** Ibrahim A. Naqid, Jonathan P. Owen, Ben C. Maddison, Anastasios Spiliotopoulos, Richard D. Emes, Andrew Warry, Monika A. Tchórzewska, Francesca Martelli, Rebecca J. Gosling, Robert H. Davies, Roberto M. La Ragione, Kevin C. Gough

**Affiliations:** 1 School of Veterinary Medicine and Science, The University of Nottingham, Sutton Bonington Campus, College Road, Sutton Bonington, Leicestershire, LE12 5RD, UK; 2 ADAS UK, School of Veterinary Medicine and Science, The University of Nottingham, Sutton Bonington Campus, College Road, Sutton Bonington, Leicestershire, LE12 5RD, UK; 3 Advanced Data Analysis Centre, The University of Nottingham, Sutton Bonington Campus, College Road, Sutton Bonington, Leicestershire, LE12 5RD, UK; 4 Animal and Plant Health Agency, Woodham Lane, New Haw, Addlestone, Surrey. KT15 3NB, UK; 5 School of Biological Science, Royal Holloway, University of London, Egham, Surrey. TW20 0EX, UK; 6 School of Veterinary Medicine, Daphne Jackson Road, University of Surrey, Guildford, Surrey. GU2 7AL, UK

## Abstract

Mapping polyclonal antibody responses to infectious diseases to identify individual epitopes has the potential to underpin the development of novel serological assays and vaccines. Here, phage-peptide library panning coupled with screening using next generation sequencing was used to map antibody responses to bacterial infections. In the first instance, pigs experimentally infected with *Salmonella enterica* serovar Typhimurium was investigated. IgG samples from twelve infected pigs were probed in parallel and phage binding compared to that with equivalent IgG from pre-infected animals. Seventy-seven peptide mimotopes were enriched specifically against sera from multiple infected animals. Twenty-seven of these peptides were tested in ELISA and twenty-two were highly discriminatory for sera taken from pigs post-infection (P < 0.05) indicating that these peptides are mimicking epitopes from the bacteria. In order to further test this methodology, it was applied to differentiate antibody responses in poultry to infections with distinct serovars of *Salmonella enterica*. Twenty-seven peptides were identified as being enriched specifically against IgY from multiple animals infected with *S.* Enteritidis compared to those infected with *S.* Hadar. Nine of fifteen peptides tested in ELISA were highly discriminatory for IgY following *S.* Enteritidis infection (p < 0.05) compared to infections with *S.* Hadar or *S.* Typhimurium.

Understanding the antibody-mediated recognition of pathogens upon infection is paramount in revealing immuno-protective responses in the host. Mapping B cell epitopes underpins sero-diagnostics and also the development of effective vaccines. The latter can include the identification of protective epitopes for vaccine design and also the assessment of more conventional vaccines (killed or attenuated pathogens) for their efficacy in generating responses against such epitopes. However, the mapping of antibody responses to infection is not straightforward, such responses are extremely complicated with polyclonal antibodies recognising a wide range of epitopes, not all of which correlate with protection against the pathogen. Indeed, pathogens often employ the production of immunogenic components that are not involved in pathogenic processes to produce immunological responses that do not affect pathogenesis^1^. Conventional screening for infection-specific epitopes often involves the resolution of pathogen proteins on 2D SDS-PAGE gels, western blotting with polyclonal sera and the identification of recognised proteins, for example by mass spectrometry methods or microsequencing^2^,^3^,^4^. However, this method is not particularly sensitive and the resolving power of the method is also limited. Therefore, often only relatively few epitopes are identified. Bacteriophage display of peptides provides libraries of millions to billions of distinct peptides to probe antibody responses to infection. The methodology links the genotype and phenotype of the peptides as each phage displays multiple copies of a peptide on its surface and contains the concomitant gene for the peptide within its genome. The display system allows the isolation of a particular peptide based on its binding activity to an antibody and in parallel the corresponding gene is also isolated. During traditional phage display methods the peptide library is propagated in bacteria and then bound to antibody that is usually immobilised on a solid support. The majority of non-binding phage are washed away and the bound phage are then eluted, usually by a shift in pH. A panning experiment generally includes several iterative rounds of binding-washing-elution steps. In between rounds, the sub-library of phage particles is again propagated within bacteria. Individual phage clones are then randomly selected and screened in a monoclonal phage assay, usually an ELISA. Any clones that display binding are then subjected to Sanger sequencing of the individual peptide genes. Peptide phage display has most often been applied to the epitope mapping of monoclonal antibodies^5^ and can be used to reveal epitopes recognised by disease-specific monoclonal antibodies, which can then be used to develop serological assays to detect infection. Conventional phage display techniques have also been applied to mapping the immunodominant epitopes of polyclonal sera. One of the most comprehensive examples of this evaluated responses in chickens immunised with the ectoparasite *Rhipicephalus (Boophilus) microplus*^6^. This study revealed eight consensus motifs. Other examples include the identification of 4 immunodominant epitopes within ricin following immunisation of rabbits^7^ and the identification seven peptides recognised during infection with *Mycobacterium leprae*^8^. Bachler and co-workers also applied phage display panning of a random peptide library to map the polyclonal antibody response to a multicomponent HIV vaccine and compared the epitopes recognised by antibodies from protected individuals to those without protection. They succeeded in mapping a neutralising epitope to a region within one of the vaccine peptides^9^. In an alternative phage display approach, the genome of *Salmonella* Typhimurium was displayed on phage and peptides that bound to antibodies from infected pigs were selected. This identified 58 peptides and 5 were produced as recombinant proteins and were recognised by the sera of infected individuals in an ELISA^10^.

However, conventional phage display panning strategies can often fail to yield any specific ligands and this is likely due to the presence of so-called parasitic phage clones^10^,^11^,^12^,^13^,^14^,^15^,^16^ and the fact that there is always a population of background phage that are not removed by washing. Parasitic phage are phage-peptide clones that are enriched through the panning experiment but do not bind to paratopes of the antibodies. They may bind to other non-paratope regions of the antibodies, the blocking agent or the solid support^11^,^12^,^13^,^14^,^15^,^16^. Further parasitic phage may be a consequence of the vast diversity of the peptide libraries, they may have a growth advantage within the bacterial propagation steps and outcompete target-specific phage during bacterial growth^11^,^12^,^13^,^15^. The screening of just a few hundred phage-ligand clones by ELISA may not identify target-specific peptides as these can be relatively rare clones due to being out competed by parasitic phage or being swamped by non-enriched background phage.

To overcome these limitations of phage display systems, several recent reports use next generation sequencing platforms to sequence sub-libraries from panning experiments, a method termed next generation phage display (NGPD)^11^,^14^,^15^,^16^,^17^,^18^. The experiments sequence between approximately 3000 and a million sequencing reads per panning round covering vastly more peptides than conventional ELISA screening. The method has identified target-specific ligands even after a single round of panning^11^. Ligand enrichment has been identified by either comparing the frequency of ligand isolation after a panning round with the original library^11^,^15^ and/or looking for common motifs within enriched ligands^15^,^16^,^17^,^18^. Overall, these studies have demonstrated that the NGPD platforms can identify hundreds to thousands of enriched ligands over background and parasitic phage and this method may have significant application to mapping polyclonal antibody responses to infection. Indeed, Ryvkin and co-workers used NGPD to screen for enrichment of peptides from a random peptide-phage library against sera from HIV patients and by analysis of over 7000 unique peptides they demonstrated that some of the most enriched peptides contained motifs that mapped to the known HIV antigen, gp160^15^. Data from this study indicate that NGPD may be able to reveal epitopes/mimotopes that are recognised by disease-specific polyclonal antibodies.

Here, we applied NGPD to map polyclonal antibody responses to infections of pigs and chickens with *Salmonella enterica*. The serovars *S.* Typhimurium, *S.* Hadar and *S.* Enteritidis were chosen as model bacterial infections as they are known to elicit strong systemic antibody responses following infections and they are important zoonotic pathogens found in pigs and/or poultry. *Salmonella* infections place significant economic burdens on the pig and poultry industries due to compromised animal health, welfare and production and/or measures to mitigate the zoonotic threat. In addition, zoonotic serovars represent some of the most common foodborne pathogens affecting human health^19^. There is widespread use of vaccination against *Salmonella* infection in poultry and this has led to serological surveillance being largely discontinued^20^. In contrast, serological tests underpin the control of *Salmonella* infection in pigs and the development and uptake of vaccination has been hampered by the lack of a suitable test to differentiate infected from vaccinated animals^21^.

In the present study we screened for peptide binders to polyclonal sera of pigs infected with *Salmonella enterica* serovar Typhimurium. The method used a comparison of the binding of peptides to post-infection IgG with binding to pre-infection IgG across multiple animals allowing the use of two-proportion Z test analysis to rank specific-enrichment of peptides against IgG from infected pigs. The peptides were then ranked by how many times they were enriched against IgG from different infected animals. This mapping of the antibody-based immune response revealed a panel of peptides that were highly discriminatory for *S.* Typhimurium infection in pigs by serological ELISA. To further validate the method, it was then used to reveal multiple peptides that were bound specifically by antibodies generated upon infection of chickens with a particular *Salmonella* serovar.

## Results

### Selection of peptides recognised specifically by polyclonal antibody from *S.* Typhimurium infected pigs

A phage-peptide library containing both constrained and linear 9-residue peptides was selected against immobilised IgG from pigs infected with *S.* Typhimurium over 2 panning rounds. Round 2 of selection included binding of enriched phage-peptides to IgG from both infected and uninfected animals. The Z score analysis used an arbitrary cut-off of 2.0 to define enrichment of peptides specifically against the post-challenge IgG. The average number of sequence reads before bioinformatic processing was ~279,000 for phage-peptides bound to IgG from infected pigs and ~322,000 for binding to IgG from non-infected animals. After sifting the reads to only include those that contained the AEGEF and DPAKAA phagemid motifs flanking a minimum of 4 amino acids and that also had no more than 2 mismatched bases in the barcode sequence, the average numbers of peptide reads were ~18,000 and ~30,000 for panning against the infected and non-infected cohorts, respectively. An average of ~2,600 distinct peptides were analysed for binding to IgG from each infected animal.

**Figure 1:**
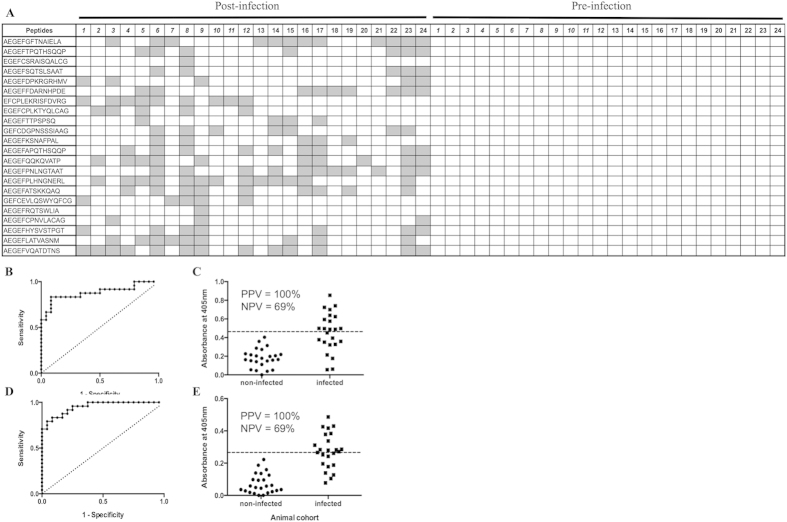
Screening of peptides specific for *S*. Typhimurium infection in pigs. Peptides identified *in silico* as being enriched against IgG from pigs experimentally infected with *S.* Typhimurium were assessed for binding to sera antibodies from 24 animals taken before challenge (pre-infection) and after challenge (post-infection). Synthetic peptide was immobilised on maxisorb plates and probed with the sera, bound antibody was then detected with an anti-porcine-IgG-AP conjugate. All samples were analysed in duplicate, ELISA signals after 2 hours incubation with substrate were taken. Binding was defined by applying a cut-off value for each peptide that was determined as the mean +3SD of the signals for the 24 non-infected samples (dashed line in **C**,**E**). ROC analysis for each peptide revealed 22 peptides (out of 27) with associated p values < 0.05 indicating that they are highly discriminatory for sera from infected pigs. Binding of sera samples to these 22 peptides is shown (**A**) as being positive (grey boxes) or negative (white boxes). Animal numbers are shown and those in italics provided sera samples for the phage panning. Analysis of the 22 peptides showed binding to all 24 sera samples from infected pigs but not to any sera from non-infected pigs. Examples of binding of individual peptides are also shown for AEGEFVQATDTNS (**B,C**) and AEGEFPLHNGNERL (**D,E**) and both bound to 13 of the 24 samples from infected pigs. ROC analysis is shown (**B,D**) along with the ELISA signals for each of the 24 infected and non-infected animals binding to peptides (**C,E**). These peptides had 100% specificity and 54% sensitivity, the positive and negative predicted values are shown for the samples tested.

In total, 77 peptides were selected as binding to IgG from between 2 and 10 of the 12 pigs. 27 of the most commonly enriched peptides representing diverse sequences were selected for further analysis (see Supplementary Information: results and discussion ‘Selection of peptides using NGS data’, and Table S3). The peptides were synthesised and tested in ELISA against sera from 24 pigs, with sera taken both before and after challenge with the pathogen (examples are given in Fig. 1). ROC curve analysis demonstrated that 22 of the 27 peptides tested were highly discriminatory for sera from infected pigs (P < 0.05, Table 1). The control peptide did not discriminate the two cohorts of sera. These 22 peptides are specific for antibodies generated upon *S.* Typhimurium infection and should therefore mimic pathogen epitopes and be of value for diagnosing infection in serological assays. To give an example of how this could be achieved, data is also displayed as a multi-peptide serological test for infection (Fig. 1) using the 24 pre-challenge samples to determine a cut-off for the assay (mean absorbance +3SD) allowing a binary output of infected or non-infected to be recorded (an analogous assay to that reported by Alban and co-workers^22^). One or more of the peptides were detected above the assay cut-off value by sera from all 24 pigs after infection but none of the sera taken before infection. Assuming that these samples are representative of immune responses in infected and uninfected animals, the assay had 100% sensitivity and specificity. In this assay, the most discriminatory individual peptides were peptides AEGEFPLHNGNERL and AEGEFVQATDTNS and both bound to 13 of the 24 sera samples from infected pigs but no sera samples from uninfected pigs (Fig. 1). These peptides individually had 100% specificity and 54% sensitivity. The 77 peptide sequences enriched in multiple animals were searched for within the SAROTUP and PepBank databases^11^ and none of the peptides had high similarity to any other peptide indicating that they are not ‘target unrelated peptides’ on parasitic phage. To further characterise the specificity of the peptides, of the 22 peptides that were highly diagnostic for *S.* Typhimurium infection in pigs (Table 1), 10 were selected at random to determine if they were also diagnostic for the same infection in chickens. Pure IgY was used in the ELISAs. When assayed against 16 IgY samples from *S.* Typhimurium infected chickens and 20 IgY samples from uninfected chickens (pre-infection samples from the 16 *S.* Typhimurium infected birds and 4 other non-infected birds), all ten peptides showed at least a strong trend for binding preferentially to the IgY from infected birds (P < 0.09, Supplementary Information Table S5). Six of the peptides demonstrated significant differential binding (P < 0.05). The control peptide did not discriminate the two cohorts of sera. The data confirms that peptides are discriminatory against *S.* Typhimurium infections and that the mapping of epitopes to infection can provide candidate diagnostic peptides to the same infection in different host species.

### Mapping polyclonal antibody responses that are specific to a particular *Salmonella* serovar

**Figure 2:**
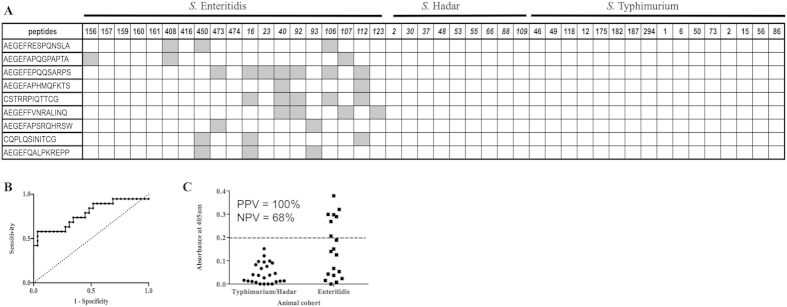
Screening of peptides specific for *S*. Enteritidis infection in chickens. Peptides identified *in silico* as being enriched against IgY from chickens experimentally infected with *S.* Enteritidis compared to birds infected with *S.* Hadar were assessed for binding to IgY from 19 birds infected with *S.* Enteritidis, 9 infected with *S.* Hadar and 16 infected with *S.* Typhimurium. Synthetic peptide was immobilised on maxisorb plates and probed with the IgY, bound antibody was then detected with an anti-IgY-AP conjugate. All samples were analysed in duplicate, ELISA signals after 2 hours incubation with substrate were taken. Binding was defined by applying a cut-off value for each peptide that was determined as the mean +3SD of the signals for the 24 non-infected samples (dashed line in **C**). ROC analysis for each peptide revealed 9 peptides (out of 15) with associated p values < 0.05 indicating that they are highly discriminatory for sera from *S.* Enteritidis infected chickens. Binding of IgY samples to these 9 peptides is shown (**A**) as being positive (grey boxes) or negative (white boxes). Animal numbers are shown and those in italics provided IgY for the phage panning. The most discriminatory individual peptides was AEGEFEPQQSARPS that bound to 7 of the 19 samples from *S.* Enteritidis infected chickens. ROC analysis is shown (**B**) along with the ELISA signals (**C)**. This peptide had 100% specificity and 37% sensitivity, the positive and negative predicted values are shown for the samples tested.

It was then assessed whether the NGPD method could map differences in antibody responses to distinct serovars of *Salmonella*, specifically to discover mimotopes of *S.* Enteritidis. In an initial round of selection, the phage-peptide library was panned against pure IgY from 9 chickens that had been infected with *S.* Enteritidis. Round 2 was against the same IgY and also against IgY from 9 chickens infected with *S.* Hadar. Again, the Z score analysis used a cut-off of 2.0 to define enrichment of peptides specifically against *S.* Enteritidis. The average number of sequence reads before bioinformatic processing was ~174,000 for phage-peptides bound to IgY from *S.* Enteritidis infected chickens and ~197,000 for binding to IgY from *S.* Hadar infected chickens. After sifting the reads to only include those that contained the AEGEF and DPAKAA phagemid motifs flanking a minimum of 1 amino acids and that also had no mismatched bases in the barcode sequence, then the average numbers of peptide reads were ~66,000 and ~74,000 for panning against the *S.* Enteritidis and *S.* Hadar infected cohorts, respectively. An average of ~7,000 peptides were analysed for binding to IgY from each animal. Twenty-seven peptides were enriched against IgY from multiple infected animals (2 chickens) and 15 were selected for further analysis (see Supplementary Information: results and discussion ‘Selection of peptides using NGS data’ and Table S4). Binding of IgY to these peptides in an ELISA was carried out using IgY from 19 chickens infected with *S.* Enteritidis, 9 chickens infected with *S.* Hadar and 16 chickens infected with *S.* Typhimurium (Table 2). ROC analysis showed that 9 of these peptides were highly discriminatory for binding to IgY from *S.* Enteritidis infected chickens (p < 0.05). The control peptide did not discriminate the two cohorts of IgY. Again, none of the 27 peptides were found within the SAROTUP and PepBank database^11^ indicating that they are not ‘target unrelated peptides’. An example of how these 9 peptides may be used to diagnose *S.* Enteritidis infection in a multi-peptide serological test is shown (Fig. 2). One or more of the peptides were detected above the assay cut-off value by IgY from 13 out of 19 chickens infected with *S.* Enteritidis. Assuming that these samples are representative of immune responses in infected animals, the assay had 68% sensitivity and 100% specificity. In this assay, the most discriminatory individual peptide was AEGEFEPQQSARPS that had 100% specificity and 37% sensitivity (Fig. 2).

## Discussion

The mapping of polyclonal antibody responses to identify individual infection-specific epitopes is vital for the diagnosis of infectious diseases and the design of effective vaccines. However, as mentioned the mapping of polyclonal antibody responses is not easy to accomplish as they are extremely complex, often involving the generation of a wide range of distinct antibodies against a diverse range of pathogen epitopes.

The advent of NGPD has provided a highly efficient screening method for phage-ligands that can succeed in identifying the enrichment of specific phage-peptides even when a low number of panning rounds are performed. The NGPD method is now being applied to the identification of epitopes in polyclonal sera. With regard to infectious diseases, in a very recent study, Xu *et al.* demonstrated the application of NGPD to map B-cell responses to viral infections^23^. They used a bespoke T7 phage-library displaying overlapping 56mer peptides covering the entire proteomes of known human viruses. They applied NGPD to map whether human polyclonal antibodies enriched peptides from a virus. The method, termed VirScan, could show high correlation between virus sequence enrichment during phage panning and data from established serological assays. However, when assaying human populations it was noted that the detection of some viruses was much lower than expected from epidemiological or vaccination data. The study noted possible reasons for this discrepancy, namely the method is limited to the display of protein epitopes and will not represent other antigenic macromolecules, it will not represent discontinuous epitopes made up of combinations of regions beyond the 56mer peptides and will not represent post-translational modifications of proteins. However, the study does comprehensively demonstrate that NGPD can be used to map epitopes within polyclonal sera.

In the present study, sera from pigs infected with *S.* Typhimurium were probed with an unbiased nonapeptide phage library containing both constrained and linear peptides. The panning strategy compared phage-peptide binding to polyclonal antibody samples from each of 12 pigs taken before and after infection and used a two-proportion Z test analysis to define specific enrichment of a particular peptide against an IgG sample from an infected animal. Peptides were then ranked in terms of how many times they were enriched against polyclonal antibody from different infected animals. 77 peptides were enriched against sera from multiple animals and 27 of these peptides that represented diverse peptide sequences were synthesised and tested in ELISA against sera from 24 pigs (pre- and post-infection). ROC analysis demonstrated that 22 of these peptides were highly discriminatory for sera from infected pigs (P < 0.05, Table 1). Eighty-one percent of the peptides tested were highly discriminatory for infection in ELISAs and none of the 77 peptides were seen in the SAROTUP or PepBank databases. Together, this strongly indicates that this panning and bioinformatic sifting strategy is highly selective for target-specific peptides and effectively dismisses parasitic phage, which can be a significant issue in phage-display panning^10^,^11^,^12^,^13^,^14^,^15^,^16^.

The focus of the study was to demonstrate the mapping of polyclonal antibody responses to infection and not to develop novel diagnostics. However, an example is given of how the 22 peptides could be used as a multi-peptide serological assay for infection and produced a test with 100% sensitivity and specificity. The mapping of individual mimotopes representing bacterial epitopes should allow effective multi-peptide assays to be developed and could be applied in array formats or as mixed peptides or as conjugated peptides^24^. Whilst it is likely that these short peptide mimotopes will provide limited information of the bacterial antigen, this is not required for them to be valid in diagnosing infection. In terms of current diagnostic assays for *Salmonella* infections, serological ELISA assays are based on a mixture of O-antigens (lipopolysaccharide) or total cell lysate as antigen and result in cross reactivity with other bacteria^25^ and also yield large differences in sensitivities between different assays^26^. The use of peptide mimotopes in a multi-peptide format may well offer significant advantages.

In a second application of the NGPD method, we probed the B-cell responses in chickens to infection with different *Salmonella* serovars in order to determine whether peptide mimotopes could be identified that were specific to infection with *S.* Enteritidis. This represents the mapping of a more subtle difference in polyclonal antibody responses as it is well established that *Salmonella* infections elicit antibody responses that can show high cross-reactivity between serovars and particularly between *S.* Enteritidis and *S.* Typhimurium^27^. The screening approach aimed to select for peptides specifically enriched against 9 chickens infected with *S.* Enteritidis compared to 9 chickens infected with *S.* Hadar. Twenty-seven peptides were enriched against IgY from multiple *S.* Enteritidis-infected animals and 15 were selected for further analysis. Binding of IgY to peptides in an ELISA was carried out using IgY from 19 chickens infected with *S.* Enteritidis, 9 chickens infected with *S.* Hadar and 16 chickens infected with *S.* Typhimurium. ROC analysis showed that 9 of these peptides were highly discriminatory for binding to IgY from *S.* Enteritidis (p < 0.05). Again, the data demonstrate that the NGPD method coupled with statistical ranking of peptide enrichment allows the selection of peptides enriched in multiple animals and that this is a highly efficient method for selecting peptides that are specific for a particular immune response. In this case 60% of the screened peptides were highly discriminatory. Some synthetic peptides may not show discrimination due to changes in their conformation once they are no longer displayed on phage or they may simply not bind to the plastic support. Both issues could be overcome by testing phage clones after recovery from the polyclonal phage sub-library after panning^28^. The identification of a range of mimotopes that can differentiate infections with different *Salmonella* serovars demonstrates the high resolution of the NGPD method.

The described method is highly efficient at mapping antibody responses to a particular infection (see Supplementary Information: results and discussion ‘Comparison of the efficacy of NGPD using random peptide libraries to other immunoproteomics methods’ and Table S6). It allows thousands of peptide-antibody binding events to be screened in parallel for each of multiple positive and negative antibody samples in a single experiment. The method uses a random peptide library that will be capable of mimicking not only linear epitopes but also conformational epitopes and those from non-protein macromolecules. In addition, the method repeatedly demonstrates that synthetic peptides identified by NGPD are suitable for high-throughput serological ELISAs. The ability to map specific polyclonal responses to infectious disease has the potential to underpin the development of novel serodiagnostics in human and veterinary medicine. It also has the potential to underpin rational vaccine design to optimise both the magnitude and duration of the immune response. It is logical that the most effective vaccines will specifically target neutralizing epitopes/mimotopes. The reported method should be applicable to the identification of a wide range of disease-specific polyclonal antibody epitopes/mimotopes as vaccine candidates and should also allow the correlation of recognition of a particular peptide with protection from clinical disease, potentially facilitating the development of highly effective vaccines. Once such epitopes are known they could also be used to determine the most effective routes and time points for administration of a particular vaccine.

## Methods

### Animal challenge studies

The animal procedures were conducted at the APHA under the jurisdiction of, and in accordance with, a UK Home Office project licence (Animals Scientific Procedures Act, 1986 that were amended in January 13 by Directive 2010/63/EU). All studies were approved by the local APHA Ethics Review Committee. For the porcine studies, twenty-four commercial piglets that were confirmed free of *Salmonella* were orally challenged with *S*. Typhimurium SL1344nal^r^ (~1 × 10^8^ cfu in 10 ml of 0.1 M pH 7.2 PBS) at 42 days old. Approximately 45 minutes prior to the challenge pigs were orally dosed with 10% sodium bicarbonate to neutralise the stomach acid (20 ml). Single blood samples were taken from each pig 10 days prior to challenge and 10 days after the *Salmonella* challenge. All blood samples were taken from the cranial vena cava using a hypodermic needle and non-heparinised vacutainer and then incubated at ambient temperature for 2 hours to allow clotting. Samples were centrifuged at 4300 g to collect the sera which was stored at −20 °C until analysis. All details of the pig challenge experiments are described elsewhere^29^.

For the poultry studies, chickens were from several challenge experiments that were conducted following the same protocol but differed slightly in terms of the age of birds at sampling and challenge. For *S.* Typhimurium challenged cohort 1 (6 birds), *S.* Enteritidis challenged cohort 1 (9 birds) and *S.* Hadar challenged birds (9 birds). Commercial Hy-line layer chickens were used and pre-infection bleeds were taken from the wing vein at 123 days old. Prior to challenge, all birds were cloacally swabbed to confirm that they were negative for *Salmonella*. Feed was withdrawn for 8 hours, the challenge strain was then administered by oral gavage at 126 days old. Prior to challenge each bird received 2 ml of 10% sodium bicarbonate to neutralise the crop acid. Following neutralisation of the crop acid each bird received 1 ml of approximately 1 × 10^9^ colony forming units (cfu)/ml of the *Salmonella* challenge strain by oral gavage. Post-infection bleeds were taken at 150 days old. The strains used for challenge were a nalidixic acid resistant *S*. Hadar, a nalidixic acid resistant *S*. Typhimurium and a nalidixic acid resistant *S*. Enteritidis PT4.

For the *S.* Enteritidis challenged cohort 2 (10 birds). Pre-infection bleeds were taken at 91 days old, challenge was at 98 days old and post-infection bleeds were taken at 112 days old. The strain used for challenge was a nalidixic acid resistant *Salmonella* Enteritidis PT4. For the *S.* Typhimurium challenged cohort 2 (2 birds). Pre-infection bleeds were taken at 138 days old, challenge was at 139 days old and post-infection bleeds were taken at 152 days old. For the *S.* Typhimurium challenged cohort 3 (8 birds). Pre-infection bleeds were taken at 124 days old, challenge was at 140 days old and post-infection bleeds taken at 153 days old. Challenge cohorts 2 and 3 used a fully sensitive *Salmonella* Typhimurium DT8. For other *Salmonella*-free birds (animal numbers 114, 146, 157 and 195), bleeds were taken at 127 days old.

### Purification of IgG and IgY

Porcine IgG was purified using a Melon™ Gel IgG Purification Kit (Thermo-Scientific,UK) following the manufacturer’s instructions. After purification of sera (50 μl) the eluate (500 μl) was stored at 4 °C after the addition of NaCl to a final concentration of 100 mM. Protein content was estimated using a Bradford reagent (Sigma-Aldrich, UK). Chicken IgY was purified by Thiophilic gel chromatography (Thermo-Scientific, UK), exactly as described previously^30^, eluted fractions were assayed by ELISA using Anti-Chicken IgY−Alkaline Phosphatase (AP) antibody produced in rabbit (Sigma-Aldrich, UK) and fractions containing IgY were pooled, protein content was estimated using a Bradford reagent (Sigma-Aldrich, UK) and samples stored at 4 °C.

### Panning of phage-peptides against purified antibody

Two peptide phage display libraries were used for biopanning experiments (kindly provided by Professor Franco Felici, University of Molise, Italy). Both libraries are based on the PC89 phagemid vector^5^,^31^ with a nonapeptide inserted at the N-terminus of the phage major coat protein pVIII. One displayed linear peptides, the other displayed constrained (cyclic) peptides where peptides are flanked by two Cys residues and therefore form a disulphide bond (see Supplementary Information: results and discussion, ‘the selection of phage peptide display libraries’ for a discussion on different phage display systems). These libraries have a diversity in the region of 10^7^ distinct peptides, NGS data indicates that 95% of clones display a unique peptide and the bias in amino acid composition is similar to that described for other phage display libraries (see Supplementary Information: results and discussion, ‘Determining bias in the display of peptides on pVIII’, Fig. S2).

Library propagation, manipulation and phage rescue was carried out in *Escherichia coli* TG1 supE thi-1 ∆(lac-proAB) ∆(mcrB-hsdSM)5(rK–mK) (F ´traD36 proAB lacIqZ∆M15). The two phage libraries were propagated separately and then pooled as equal amounts of phage.

**Figure 3:**
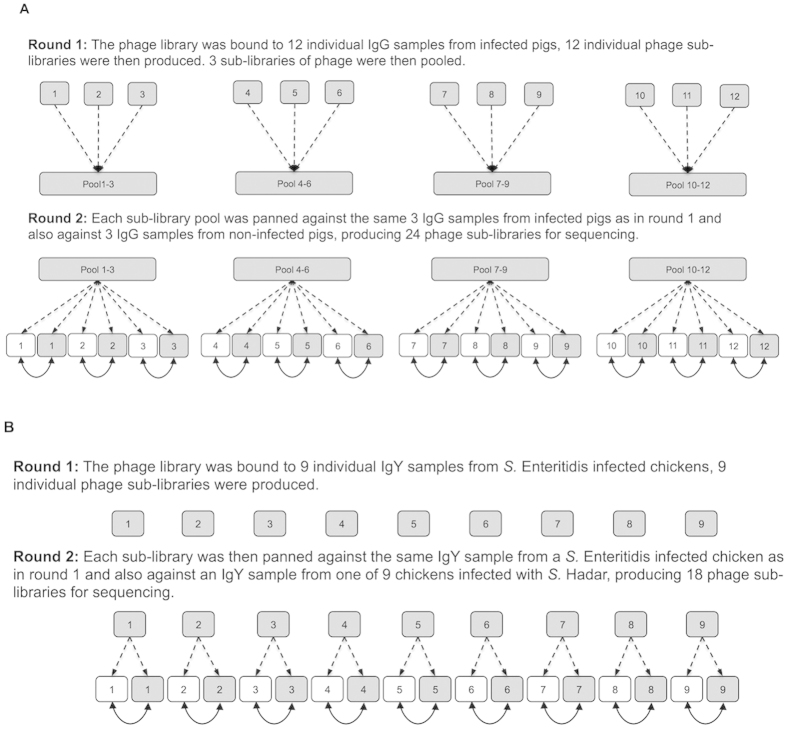
Panning of peptide libraries against purified polyclonal IgG **(A)** or IgY **(B).** Panel A: Phage libraries were bound in parallel to 12 IgG samples from pigs infected with *S.* Typhimurium and after elution these 12 sub-libraries of phage were propagated and groups of 3 were pooled (round1, dashed arrows) in equal phage numbers. Each sub-library pool was then bound to IgG from the same 3 animals used in round 1 this time using IgG taken post-challenge (grey boxes) and IgG taken pre-challenge (white boxes) with the pathogen (round 2, dashed arrows). Bioinformatic analysis to assess for enrichment of individual peptides specifically against IgG from infected animals was carried out by comparing the binding of peptides to IgG taken pre- and post-infection with the pathogen (solid arrows). Panel B: Phage libraries were bound in parallel to 9 IgY samples from chickens infected with *S.* Enteritidis and after elution these 9 sub-libraries of phage were propagated. Each sub-library was then bound to IgY from the same *S.* Enteritidis-infected chicken used in round 1 (grey boxes) and also against IgY from one of nine chickens infected with *S.* Hadar (white boxes) as indicated by dashed arrows. Bioinformatic analysis to assess for enrichment of individual peptides specifically against IgY from *S.* Enteritidis-infected animals was carried out by comparing the binding of peptides to IgY from *S.* Enteritidis-infected birds with those infected with *S.* Hadar (solid arrows).

Libraries were panned against IgG from each of 12 pigs infected with *S.* Typhimurium largely as previously described^32^ and as outlined in Fig. 3A. Briefly, each of the libraries were grown to mid-log phase in TG1 *E. coli*, superinfected with M13KO7 helper phage and incubated overnight at 30 °C (250 rpm) to produce phage particles. The two libraries were then titred and mixed in a 1:1 ratio of phage in PBS and 3% (w/v) marvel, and incubated at room temperature for 2 h. IgG (20 μg/ml) was immobilised overnight in maxisorp plastic microtitre wells (100 μl per well, 10 wells per sample). Following washing, wells were blocked with 3% (w/v) marvel for 1 h at room temperature. Phage (~4 × 10^11^/ml, 300 μl/well) were then bound to the immobilised antibodies for 2 h at room temperature, phage solution was then removed and the phage binding step repeated with fresh phage three more times with the final binding step carried out for 16 h. Wells were then washed 20x with each of PBS (10 mM phosphate buffer pH 7.2–7.4 with 150 mM NaCl) plus 0.1% (v/v) Tween (PBST) and then 20x with PBS. Bound phage were eluted by competition for binding using 1 mg/ml of *Salmonella* lysate (using the target serovar, 100 μl/well; produced as previously described^29^) incubated for 45 min at room temperature. Eluted phage from each antibody sample (10 wells) were pooled and used to infect TG1 cells and grown on solid media with ampicillin selection (2YT plus 150 μg/ml ampicillin). The cells were then harvested and used to propagate a sub-library of phage as before. Sub-libraries of phage were produced from the panning against antibody from each of the 12 animals. When panning against pig IgG, these were pooled into 4 libraries (phage from 3 phage sub-libraries were pooled together). A second round of panning was carried out exactly as the first round except that a phage pool selected against IgG from 3 particular animals was bound to both IgG taken post-challenge (4 wells) and pre-challenge (4 wells) with the pathogen for these same three animals. Panning steps were the same as in round 1 except washing was 20x in PBST-BSA (0.1% Tween 20, 500 μg/ml BSA, and wash solution incubated in wells for 2 min for each wash) and 20x in PBS. Again, eluted phage isolated against an individual pre-challenge or post-challenge sample was pooled and used to infect TG1 cells. Following growth of the infected TG1 on 2YT plates with ampicillin the cells were harvested and stored at −80 °C in 30% (w/v) glycerol.

Panning was also performed to discover peptides specific for IgY from chickens infected with *S.* Enteritidis, this was carried out in the same way with minor alterations and as outlined in Fig. 3B. Round one of panning used (~1 × 10^12^/ml, 300 μl/well) and was against 9x IgY samples from chickens challenge with *S.* Enteritidis (challenge cohort 1). These sub-libraries of phage were individually propagated for round 2 of panning where each sub-library was bound to the same post-challenge IgY sample as in round 1 and also one of nine IgY samples from chickens challenged with *S.* Hadar.

### DNA extraction and sample preparation for Ion Torrent sequencing

Amplicon containing the peptide gene was produced (Supplementary Information Figure S1). Bacterial glycerol stocks were grown overnight in 2YT with ampicillin and the phagemid DNA extracted using QIAprep® Spin Miniprep Kit (QIAGEN Ltd, UK). A primer set specific to PC89 vector sequences that flanked the peptide gene and amplified a 330 bp fragment was used in a PCR reaction to amplify the peptide gene sequences, these primers also contained a linker sequence (Supplementary Information Table S1, P8 forward and reverse primers). PCR products were purified using the NucleoSpin Gel and PCR Clean-up kit (Macherey-Nagel). Purified product (1 μl) was amplified with a second primer set (Supplementary Information Table S1, Akey-BC-linker1(Forward) and P1prim-linker2(Reverse) primers) that included a distinct barcode (Supplementary Information Table S2) sequence and A and P1 adapter sequences designed for the Ion Torrent sequencing platform.

Samples were multiplexed (50 different barcoded primers were available; see Supplementary Information Table S2) in each sequencing run. The PCR products for each barcode were quantified by Qubit ds DNA BR Assay Kit and pooled in equal amounts and resolved by agarose gel electrophoresis and the band of interest (~330 bp) was purified using the NucleoSpin Gel and PCR Clean-up. A second purification of the amplicons was performed with an Agencourt®AMPure®XP PCR purification kit (Beckman Coulter, UK) following the manufacturer’s protocol, and the amplicon was then quantified as before. DNA amplicon (a minimum of 50 μl of 100–200 ng DNA) was sequenced via an Ion Torrent PGM service (University of Pennsylvania, US) on a 318 chip.

### NGS data analysis

Perl scripts were used to process NGS data files. Scripts used for data processing are provided at http://figshare.com/articles/Mapping_B_cell_responses_to_bacterial_infection_using_next_generation_phage_display/1566818. Briefly, FASTQ files were converted to FASTA files and Ion Torrent barcodes used in each experiment identified using the “FASTQ/A Barcode splitter” (part of the FASTX-toolkit from http://hannonlab.cshl.edu/fastx_toolkit/index.html). DNA sequences in each barcode-binned FASTA file were translated in all 3 frames and concatenated to a single file (translate.pl). FASTA files were processed to identify matching flanking sequence motifs (AEGEF and DPAKAA) to capture insert (peptide) sequence. Parameter choices for analysis of pig IgG data were as follows, maximum 2 mismatches allowed per barcode, minimum of 4 amino acid peptide between flanking motifs for analysis, amber stop codon (TAG) replaced with amino acid Q. For chicken IgY analysis a maximum 0 mismatches allowed per barcode, minimum of 1 amino acid between flanking motifs for subsequent analysis, all stop codons replaced with amino acid Q. Each peptide sequence obtained against a target antibody sample was compared to the set of peptide sequences obtained against the corresponding control antibody sample. For each peptide encoding sequence, the frequency and ratio (number of copies of the sequence isolated against the target protein: against the control protein) and the total number of peptide gene reads per sample were compared by analysing the data using a two proportion Z test:


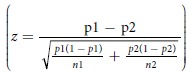


This analysis method was first applied to the analysis of gene expression data and has recently also been applied to the identification of enriched phage antibodies when binding a phage-antibody library against a target and control^18^. This Z score reflects the ratio as well as the absolute frequencies of each peptide sequence and ranks them by relative statistical importance. (n1 = number of peptide sequences obtained with the target sample, n2 = number of peptide sequences obtained with the control sample, p1 = the number of sequences obtained for a specific peptide against the target sample/n1, p2 = the number of sequences obtained for a specific peptide against the control sample/n2). Peptide sequences seen enriched above an arbitrary Z-score threshold of 2.0 were ranked based on the number of positive animals in which they were seen. The Z-test requires samples to have been drawn from independent populations. In the case where the panning experiments are pre and post infection and hence may be correlated, this may influence the power of the results. We are likely to have suffered from type II error. However, as the z-score was used only for ranking of peptides and we then empirically tested for discriminatory power we are confident that this approach provides a useful method for identifying the most promising candidate enriched peptides.

### Peptide Synthesis

Selected peptides enriched against IgG from *S.* Typhimurium infected pigs were synthesised with an amidated C-terminus and with the protein VIII N-terminal residues AEGEF (as indicated) up to a maximum peptide length of 15 amino acids (Table 1). Constrained peptides also contained a C terminal G residue. It should be noted that residues encoded by an ochre or opal stop codon were removed from the peptide sequences described here rather than including a Q that would be the case for any read through events^33^. Selected linear peptides identified by NGS as enriched against IgY from *S.* Enteritidis infected chickens were synthesised with an amidated C-terminus and with the protein VIII N-terminal residues AEGEF (Table 2). Constrained peptides isolated against IgY contained a C terminal G residue as the only vector-derived residue. Stop codons within peptide sequences were replaced by a Q in the synthesised peptides. An unrelated control peptide (HVMDADQESVSQSDI) was also synthesised for use as a control throughout these experiments.

All peptides were synthesised by Biomatik Corporation (Ontario, Canada) at the crude purity level (>49% pure) and 5 mg scale. The synthetic peptides were then dissolved and diluted to a stock concentration of 2–10 mg/ml.

### Peptide ELISA

Peptides (100 μl, 100 μg/ml) were coated in duplicate wells (Thermo Scientific™ Nunc, UK) in coating buffer (100 mM sodium carbonate-bicarbonate buffer, pH 9.6) at 4 °C overnight. Wells were washed 1x with PBST and 1x with PBS and blocked with 3% (w/v) skimmed milk powder in PBS for 1 h then washed as before. Bound protein was probed with sera (1:50 in PBS containing 3% marvel, 100 μl/well) or purified IgY (100 μl, 20 μg/ml). Bound IgG was detected using goat anti-pig IgG (Source BioScience, UK) alkaline phosphatase (AP) secondary antibody (1:4000 in PBS containing 3% marvel, 100 μl/well) and bound IgY was detected using an anti-chicken IgY- AP conjugate (Sigma-Aldrich, UK; 1:20,000 in PBS containing 3% marvel, 100 μl/well); both were then detected with 100 μl of p-Nitrophenyl Phosphate substrate (Sigma-Aldrich, UK). All wash steps after addition of antibody were 6x with PBST and 6x with PBS. Absorbance was measured at 405 nm.

### Analysis of ELISA data

All samples were analysed in duplicate and a mean calculated. For each antibody sample, there was a background control that had no immobilised peptide. The reading for this background was subtracted from all readings for that antibody sample before processing of the data. Receiver Operator Characteristic (ROC) analysis was carried out using Graph Pad Prism 6 for each peptide (including the control peptide) bound by the full cohort of sera/IgY samples in each experiment, area under the curve (AUC) and p values were generated using a 95% CI. A peptide was considered to be highly discriminatory if the p value was 0.05 or below. Examples of using discriminatory peptides in a serological assay were generated using a cut-off value for each peptide generated from the mean of the absorbance of all negative samples plus 3 standard deviations.

## Additional Information

**How to cite this article**: Naqid, I. A. *et al.* Mapping polyclonal antibody responses to bacterial infection using next generation phage display. *Sci. Rep.* **6**, 24232; doi: 10.1038/srep24232 (2016).

## Supplementary Material

Supplementary Information

## Figures and Tables

**Table 1:** ELISA screening of candidate peptides identified by NGPD as being enriched against IgG from *S.* Typhimurium infected pigs.

Peptide sequence^a^	*In silico* assessment^b^	ELISA assessment	
		AUC of ROC^c^	P value^c^
AEGEFGFTNAIELA	10/12	0.965	<0.001
AEGEFTPQTHSQQP	7/12	0.868	<0.001
EGEFCSRAI*SQALCG	7/12	0.667	0.048
AEGEFSQTSLSAAT	6/12	0.922	<0.001
AEGEFDPKRGRHMV	6/12	0.762	0.002
AEGEFFDARNHPDE	6/12	0.852	<0.001
EFCPLEKRISFDVRG	5/12	0.743	0.004
EGEFCPLKTYQL*CAG	5/12	0.710	0.013
AEGEF*TTP*SPSQ	5/12	0.760	0.002
GEFCDGPNSSSIAAG	5/12	0.832	<0.001
AEGEFKSNAFP*AL	5/12	0.832	<0.001
AEGEFP*SPKMRVW	5/12	0.637	0.103
EGEFCTHCKFALL*CG	3/12	0.619	0.158
AEGEFAPQTHSQQP	3/12	0.880	<0.001
EGEFCTCAQSLRG*CG	3/12	0.569	0.409
AEGEFQ*QKQVATP	3/12	0.946	<0.001
AEGEFPNLNGTAAT	3/12	0.948	<0.001
AEGEFPLHNGNERL	2/12	0.955	<0.001
AEGEFAKCRASKRQ	2/12	0.632	0.117
AEGEFATSKK*QAQ	2/12	0.908	<0.001
GEFCEVLQSWYQFCG	2/12	0.693	0.022
AEGEFR*QTSWLIA	2/12	0.676	0.036
EFCCTPPGKPCEVRG	2/12	0.619	0.155
AEGEFCP*NVLA*CAG	2/12	0.750	0.003
AEGEFHYSVSTPGT	2/12	0.887	<0.001
AEGEFLATVASN*M	2/12	0.899	<0.001
AEGEFVQATDTN*S	2/12	0.885	<0.001
Control peptide	–	0.581	0.338

^a^Opal and ochre codons are denoted by an asterisk. Residues coded for by these stop codons were omitted in the synthesised peptides. N-terminal residues AEGEF are phage coat protein residues. Constrained peptides also contain a phage coat protein G residue at their C-terminus.

^b^Number of IgG samples that the peptides was enriched against/total number of IgG samples: enrichment was defined using a Z score of ≥2.0.

^c^ROC curves were produced for the recognition of peptides with sera from infected (n = 24) and non-infected (n = 24) pigs in an ELISA. AUC and corresponding p values are listed.

**Table 2:** ELISA screening of candidate peptides identified by NGPD as being enriched against IgY from chickens infected with the serovar *S.* Enteritidis.

Peptide sequence^a^	*In silico* assessment^b^	ELISA assessment	
		AUC of ROC^c^	P value^c^
AEGEFRESPQNSLA	2/9	0.69	0.030
AEGEFLPRPIQPTP	2/9	0.60	0.260
AEGEFAPQGPAPTA	2/9	0.68	0.045
AEGEFPRNQEHFAM	2/9	0.60	0.260
AEGEFRSQSRRFHL	2/9	0.64	0.127
AEGEFEPQQSARPS	2/9	0.77	0.002
AEGEFAPHMQFKTS	2/9	0.67	0.050
AEGEFWARTHVQKV	2/9	0.67	0.059
CSTRRPIQTTCG	2/9	0.76	0.003
AEGEFFVNRALINQ	2/9	0.83	<0.001
AEGEFAPSRQHRSW	2/9	0.71	0.021
AEGEFQGASGRSCR	2/9	0.54	0.661
AEGEFMALLNQSYP	2/9	0.67	0.063
CQPLQSINITCG	2/9	0.79	0.001
AEGEFQALPKREPP	2/9	0.69	0.034
Control peptide		0.54	0.696

^a^For linear peptides, N-terminal residues AEGEF are phage coat protein residues. Constrained peptides contain a phage coat protein G residue at their C-terminus.

^b^Number of IgY samples that the peptides was enriched against/total number of IgY samples: enrichment was defined as peptides giving a Z score of ≥2.0.

^c^ROC curves were produced for the recognition of the peptides with IgY from *S.* Enteritidis infected (n = 19) and *S.* Typhimurium or *S.* Hadar infected (n = 25) chickens in an ELISA. AUC and corresponding p values are listed.
