# Supplementary material for: Mapping polyclonal antibody responses to bacterial infection using next generation phage display
Source: Sci Rep. 2016 Apr 13;6:24232. doi: 10.1038/srep24232 (PMC4829855; doi:10.1038/srep24232)
Supplement: Supplementary Information [file srep24232-s1.pdf]

Mapping polyclonal antibody responses to bacterial infection using next generation phage display

Ibrahim A. Naqid[a], Jonathan P. Owen[b], Ben C. Maddison[b], Anastasios Spiliotopoulos[a], Richard D. Emes[a,c], Andrew Warry[a,c], Monika A. Tchórzewska [d,e,], Francesca Martelli[d], Rebecca J. Gosling[d], Robert H. Davies[d], Roberto M. La Ragione[f] and Kevin C. Gough[a]*

Supplementary methods

Table S1. Primers used in the PCR amplification of peptide gene sequences from PC89VIII and PC89VIII-C for sequencing on the Ion Torrent platform.

Primer name	DNA sequence (5'→3')
P8-Forward1	*GTAATCCTTGTGGTATCG*GATGCTGTCTTTCGCTGC
P8-Reverse1	*CTAGAACATTTCACTTAC*GGTTTTCCCAGTCACG
Akey-BC-linker1 (Forward)	CCATCTCATCCCTGCGTGTCTCCGACTCAGCTAAGGTAAC*GTAATCCTTGTGGTATCG*[a]
P1prim-linker2 (Reverse)	CCTCTCTATGGGCAGTCGGTGAT*CTAGAACATTTCACTTAC*

[a]The barcode region is underlined and 50 variants were available to use. This region in each primer varies in at least 5 bases from any other primer. Codons in italics are linker regions that are the same in both forward primers or both reverse primers.

Table S2: Barcodes used to label PCR products.

Barcode	Sequence	Barcode	Sequence
BC01	CTAAGGTAAC	BC26	TTACAACCTC
BC02	TAAGGAGAAC	BC27	AACCATCCGC
BC03	AAGAGGATTC	BC28	ATCCGGAATC
BC04	TACCAAGATC	BC29	TCGACCACTC
BC05	CAGAAGGAAC	BC30	CGAGGTTATC
BC06	CTGCAAGTTC	BC31	TCCAAGCTGC
BC07	TTCGTGATTC	BC32	TCTTACACAC
BC08	TTCCGATAAC	BC33	TTCTCATTGAAC
BC09	TGAGCGGAAC	BC34	TCGCATCGTTC
BC10	CTGACCGAAC	BC35	TAAGCCATTGTC
BC11	TCCTCGAATC	BC36	AAGGAATCGTC
BC12	TAGGTGGTTC	BC37	CTTGAGAATGTC
BC13	TCTAACGGAC	BC38	TGGAGGACGGAC
BC14	TTGGAGTGTC	BC39	TAACAATCGGC
BC15	TCTAGAGGTC	BC40	CTGACATAATC
BC16	TCTGGATGAC	BC41	TTCCACTTCGC
BC17	TCTATTCGTC	BC42	AGCACGAATC
BC18	AGGCAATTGC	BC43	CTTGACACCGC
BC19	TTAGTCGGAC	BC44	TTGGAGGCCAGC
BC20	CAGATCCATC	BC45	TGGAGCTTCCTC
BC21	TCGCAATTAC	BC46	TCAGTCCGAAC
BC22	TTCGAGACGC	BC47	TAAGGCAACCAC
BC23	TGCCACGAAC	BC48	TTCTAAGAGAC
BC24	AACCTCATTC	BC49	TCCTAACATAAC
BC25	CCTGAGATAC	BC50	CGGACAATGGC

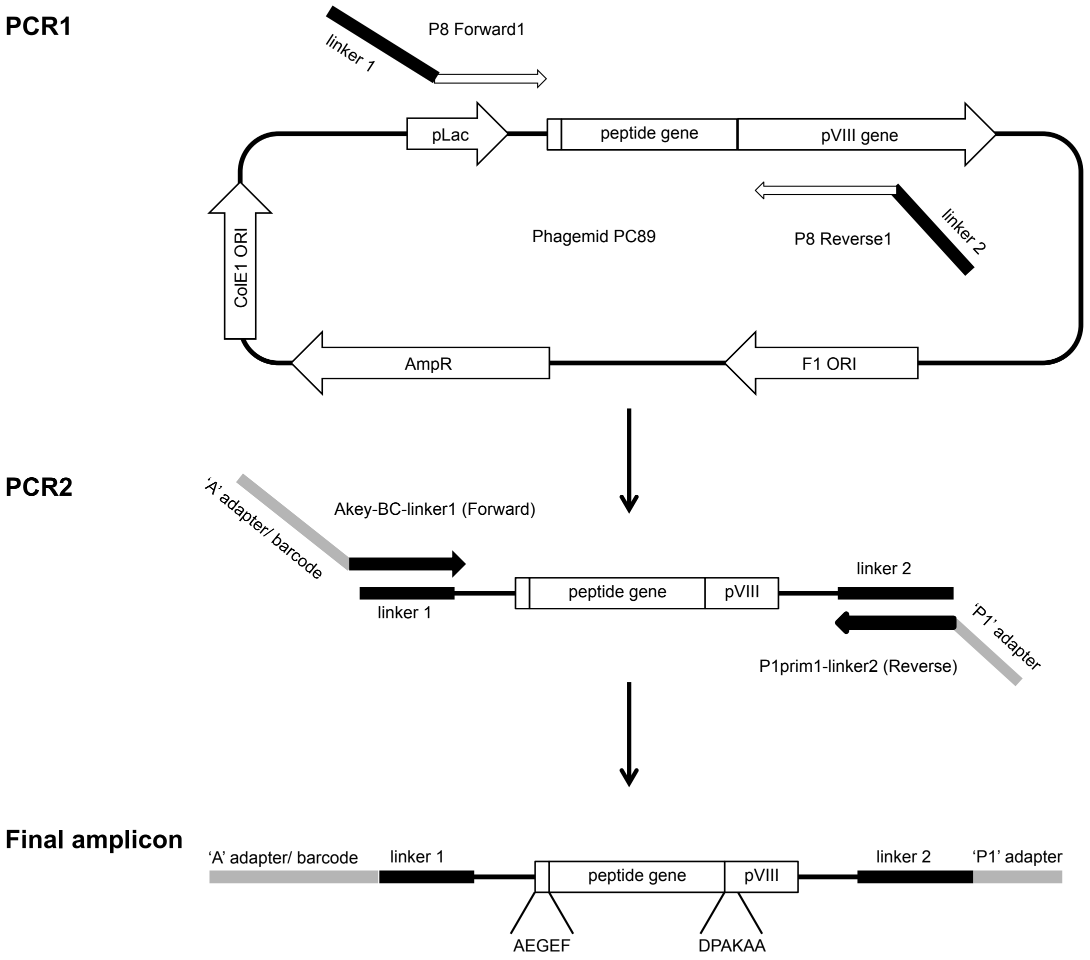

Figure S1. Schematic of the amplification of the peptide genes for NGS analysis. The primer set homologous to the PC89 vector sequences that flanked the peptide gene, P8 Forward1 and P8 Reverse1, were used to amplify a 330bp fragment (PCR1), each primer also contained a distinct linker sequence. Purified PCR1 product (1 μl) was then amplified (PCR2) with a second primer set, Akey-BC-linker1(Forward) and P1prim-linker2(Reverse). The Akey-BC-linker1(Forward) includes the linker 1 sequence along with a distinct barcode sequence and an 'A' adapter. The P1prim-linker2(Reverse) contains the linker 2 sequence and a P1-adapter (A and P1 adapter sequences are designed for the Ion Torrent sequencing platform). The motifs flanking the peptide gene are shown (AEGEF and DPAKAA). Each sample from the second round of panning was amplified in this way to tag with a unique barcode sequence and the final amplicons were then mixed for NGS analysis.

Supplementary results and discussion

The selection of phage peptide display libraries

When considering which phage-peptide display system to use there are a few issues that can be considered. Using a phage vector system for pVIII display severely restricts the size of peptide that can be displayed. It has been suggested that a limit of 6-8 residues may be due to size constraints in passing through the pIV membrane pore and/or due to interaction of the pVIII with pVII during phage particle assembly. The use of phagemid systems overcomes this limitation as the number of peptides displayed per phage particle is considerably less, up to ~200 peptides[1]. One issue pertinent to phagemid mediated pVIII display is that the peptide size and charge still influence the number of copies of the peptide displayed on the phage[2]. By comparison, there is little constraint on the size of proteins/peptides that can be display on pIII and there is usually between 1 and 5 copies of the protein/peptide per phage particle.

Peptides being displayed on phage need to be compatible with *E. coli* transcription and translation and the phage assembly and secretion processes as well as phage infection. Any negative influence of a peptide on these processes will influence the production of that phage clone. So for pIII and pVIII display systems the distinct effects of individual peptide clones on phage production may influence bacterial growth and survival. It will also affect the valency of the display which has the potential to be considerably more variable for pVIII display compared to pIII.

A further consideration is whether to use linear or constrained peptides, the latter have increased stability which can contribute to increased binding ability compared to linear peptides. Here, we used a mixture of both types of peptide to provide a comprehensive range of structures for mapping mimotopes.

Determining bias in the display of peptides on pVIII

The pC89 phage libraries used in the present study were analysed by sequencing the peptide-insert gene regions and the libraries differed considerably in the proportion of phagemid that did not contain a peptide insert and instead contained a single stop codon, 73% and 3% for the linear and constrained libraries respectively. These "empty phagemid" are due to the library construction method and was previously reported in the original manuscripts describing the production of the libraries: the linear library was reported to contain 40% of clones having no insert[3] and the

constrained library reported as having 75% of clones with no insert[4]. As reported previously, clones that do not contain insert may well have growth advantages[5], but the data here for the naïve libraries may suggest that this is only the case for linear peptide display on pVIII and actually the proportion of “empty phagemid” clone was reduced upon propagation of the constrained peptide library. However, upon selection and repeat propagation of the libraries over 3 rounds of panning the proportion of “empty phagemid” became more similar for these two library types: 21% and 31% for the constrained and linear peptide libraries, respectively. This would indicate that both library types support the propagation of “empty phagemid” and it is likely to represent a significant proportion of clones throughout panning experiments. However, when screening for phage binders by NGS such clones can easily be discarded which is not the case for conventional screening using immunoassay analysis of picked phage clones.

Peptide display libraries often represent only a small fraction of the theoretical diversity due to the practical constraints of the size of library that can be produced. There will also be bias in the peptides that are displayed. However both issues are partly overcome by the sequence plasticity of the binding peptide motifs. It is expected that display of peptides on pVIII will have similar constraints and bias to the display of peptides on pIII. Rhodi and co workers[6] stated that by far the greatest bias in peptide display is due to the degeneracy in the codon usage during library construction. The next, much smaller, influence on peptide bias is due to selection against peptides with odd numbers of C residues. This is thought to be due to aberrant disulphide bridge formation, including cross linkage of protein fusions preventing effective protein secretion and phage assembly. It is also well documented that for there is bias in residues downstream of the peptidase cleavage site. These residues, and in particular within the first 3 residues after the cleavage site, are known to be important for the positioning of the phage coat proteins within the periplasmic space for cleavage by the enzyme[2,6]. However, the libraries used in the current study have an AEGEF sequence following this cleavage site and before the peptide insert sequence. The presence of wild type sequence downstream of the peptidase cleavage site should reduce bias in displayed peptides that is imposed by variable efficacy of peptidase cleavage of the signal peptide. It is also well documented that phage display of peptides may favour those containing beta turn structures compared to alpha helices or beta sheet[6,7] meaning that P residues are over represented. In addition, It has

also been reported that peptides containing 4 or more repeating amino acid residues are not effectively displayed which may be due to protein translation inefficiencies, although the high copy number of pVIII may also contribute to this and it may not be an issue for pIII display[2].

For the pC89 libraries, when analysing the display of distinct peptides both libraries produced very similar results, in total ~123,000 insert-containing clones were analysed and of these ~95% were unique peptides, ~5% of peptides were seen twice and 0.3% were seen 3 times (Figure S2). Both libraries are estimated to contain ~10^7 unique clones[3,4], when assuming Poisson statistics and an equal probability of each peptide occurring the sequence data should show over 99% of the clones being unique. As only ~95% of clones were unique for each library, the data indicates some bias in peptide display, this data is similar to results obtained with commercial pIII libraries[8,9]. When considering the amino acid composition of the libraries (Figure S2) the use of the NNN codon in constructing the peptide library has heavily biased the peptide composition with L, R and S over-represented and M and W under-represented. When considering amino acid bias due to phage propagation, comparing the theoretical and actual amino acid compositions of the libraries shows that the most obvious differences are that P and A and stop residues (assumed to be replaced with a Q) are proportionally higher than expected in both libraries and the residues C, G, I and V are lower than expected. This is broadly similar to the previously reported data on pIII libraries, especially when considering the stop, C and P residues. Overall, the pC89 libraries present a relatively low diversity of peptides (~10^7 peptides) with likely high valency, compared to commonly used pIII libraries. Whilst there is some bias in the peptides being displayed, approximately 95% of the peptides are unique. The peptides also contain bias in terms of the frequency of the individual amino acids being displayed and this is similar to that reported for other library formats[8]. However, it should be noted that despite any limitations in the pC89 libraries and especially the relatively small repertoire of peptides on display, the reported methods were highly efficient in selecting “infection-specific” peptide mimotopes from these libraries.

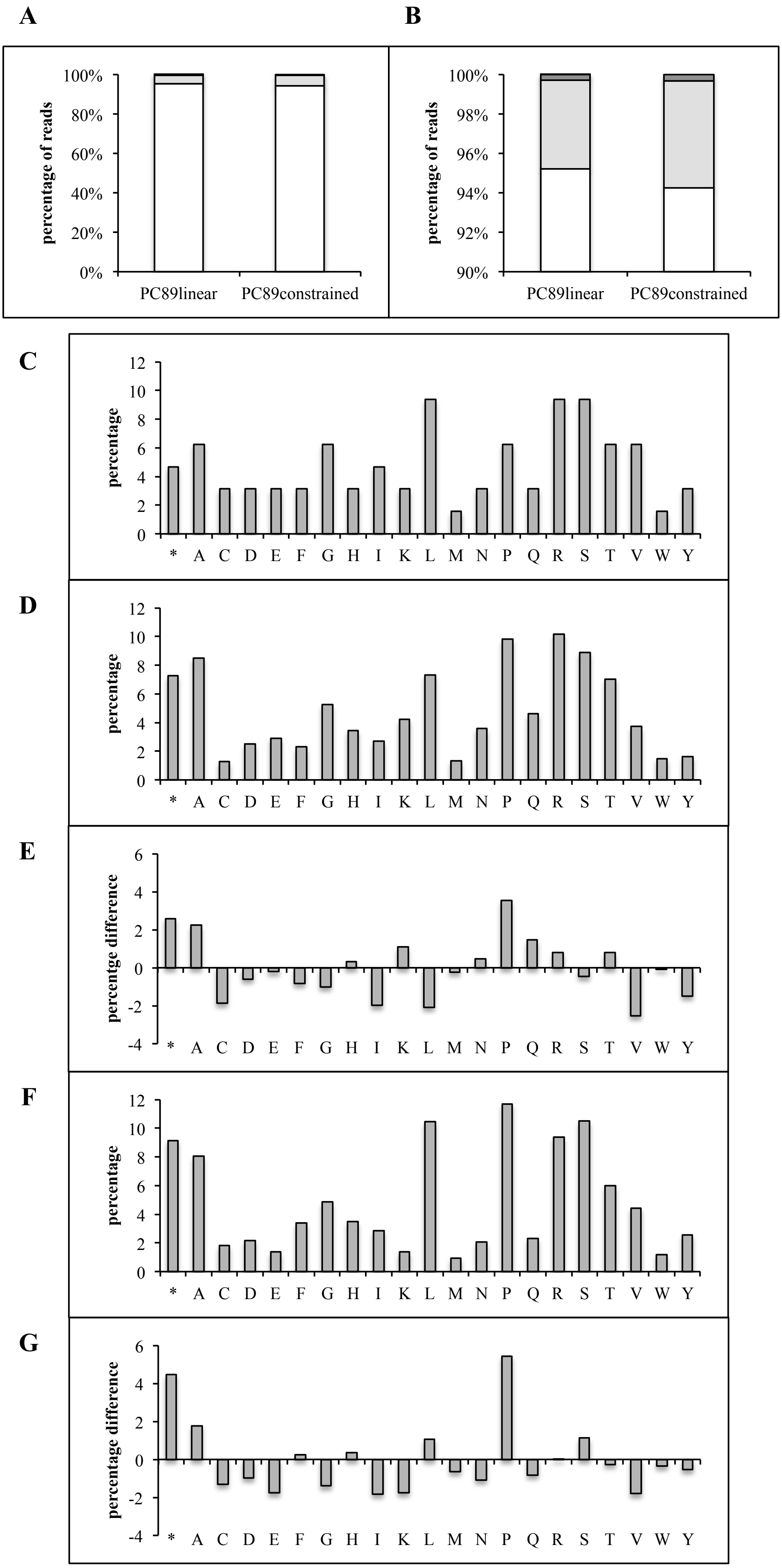
A
B
percentage of reads
100%
80%
60%
40%
20%
0%
98%
96%
94%
92%
90%
PC89linear
PC89constrained
C
D
F
percentage
E
G
percentge difference
percentage difference
* A C D E F G H I K L M N P Q R S T V W Y

Figure S2. Frequency of display of peptides (A, B) and amino acids (C-G) on pVIII for the pC89 phagemid vector system. To determine the frequency of display of distinct peptides in the naïve phage libraries, the phage-peptide genes were sequenced producing ~84,000 and ~39,000 sequencing reads for the linear and constrained pC89 phagemid libraries respectively. For the linear peptide library: 95.2% of the peptides were identified once (A and B, white), 4.5% of peptides were seen twice (A and B, light grey), and 0.3% of peptides were seen 3 times (A and B, dark grey). Two peptides were identified 4 times representing 0.009% of the peptides sequenced. No peptide was identified more than 4 times. For the constrained peptide library: 94.3% of the peptides were identified once (A and B, white), 5.4% of peptides were seen twice (A and B, light grey), and 0.3% of peptides were seen 3 times (A and B, dark grey). No peptides were identified more than 3 times. When considering the amino acid composition (%) within the phagemid libraries (C-G), the theoretical amino acid composition when using NNN codons for the library construction is shown (C). The actual amino acid compositions for the naïve linear (D) and constrained (F) peptide libraries were determined. For each amino acid, the difference between the composition percentage for the theoretical and actual values are shown for the linear (E) and constrained (G) peptide libraries.

Selection of peptides using NGS data

For the selection of peptides against IgG from *S.* Typhimurium infected pigs, analysis of NGPD data demonstrated that 77 peptides were enriched against IgG from between 2 and 10 of the 12 infected pigs (Table S3). Twenty-seven of the most commonly enriched peptides representing diverse sequences were synthesised and tested in ELISA. Selected peptides included representatives of the 4 motifs found upon analysis of all 77 peptides with the MEME algorithm[10] (Table S3). Of the 29 peptides not represented by these motifs, all peptides that were enriched against sera from more than two animals were selected (12 peptides, Table 1). Finally, some of those peptides that were enriched against 2 animals were arbitrarily selected (10 out of 17, Table S3).

For selection of peptides against IgY from *S.* Enteritidis infected chickens, analysis of NGPD data demonstrated that 27 peptides were enriched against IgG from 2 chickens (Table S4). No peptides were identified as being enriched in more than 2 chickens and no motifs were identified for these peptides upon analysis with the MEME algorithm[1].

The data generated with peptides selected against IgG from *S.* Typhimurium infected pigs demonstrated that 8 out of 10 peptides that were enriched against 2 animals and did not belong to a common motif were nevertheless specific for infection. Therefore, from the chicken data, an arbitrary selection of 15 peptides (out of the 27) was carried out for synthesis and further analysis in ELISAs.

Table S3. Peptides and associated motifs for candidate epitopes/mimotopes that interact with IgG from pigs infected with *S*. Typhimurium.

**Motif[a]**	**Contributing peptides [total number]**
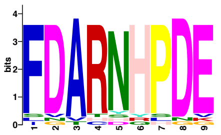	**FDARNHPDE,** FDACNHPDE, FDAHNHPDE, FDARDHPDE, FDARNHHDE, FDARNHLDE, FDARNHPDG, FDARNHPDK, FDARNHPDV, FDARNHPGE, FDARNHPNE, FDARNHSDE, FDARNQPDE, FDARNRPDE, FDARNYPDE, FDARSHPDE, FDARYHPDE, FDATHHPDE, FDTRNHPDE, FDVRNHPDE, FGARNHPDE, FNARNHPDE, FVARNHPDE, IDARNHPDE, LDARNHPDE, SDARNHPDE [26]
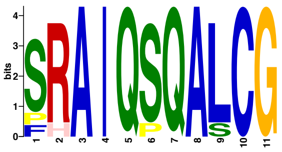	**CSRAI*SQALCG**, CFRAI*S*ALCG, CPRAI*S*ALCG, YSRAI*S*ALCG, CSHAI*S*ALCG, CSRAI*P*ALCG, CSRAI*S*ALCG, CSRAI*S*ASCG [8]
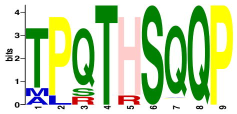	**APQTHSQQP, TPQTHSQQP,** MPQTHSQQP, TLQTHSQQP, TPQTHS*QP, TPQTHSQ*P, TPQTRSQQP, TPRTHSQQP, TPSTHSQQP [9]
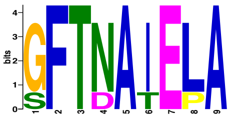	**GFTNAIELA,** GFTDAIELA, GFTNAIEPA, GFTNATELA, SFTNAIELA [5]
No motif	**DPKRGRHMV, SQTSLSAAT, *TTP*SPSQ, CDGPNSSSIAAG, KSNAFP*AL, P*SPKMRVW, CTHCKFALL*CG, CTCAQSLRG*CG, Q*QKQVATP, CPLKTYQL*CAG, CPLEKRISFDVRG, PNLNGTAAT, PLHNGNERL, AKCRASKRQ, ATSKK*QAQ, CEVLQSWYQFCG, R*QTSWLIA, CCTPPGKPCEVRG, CP*NVLA*CAG, HYSVSTPGT, LATVASN*M, VQATDTN*S,** CCTPWETV*SAG, CDGPIPPPIAAG, CTCAQSLRGNAG, CTTPRLMPLACG, CTTPRLMPPWRAG, KPGPR*LQP, CPLKTYQLDVRG [29]

[a]The MEME algorithm was used to generate motifs for the phage-peptide sequences found to be enriched against IgG from multiple pigs infected with *S*. Typhimurium. An E-value cut-off of 0.05 was used for selecting motifs and a minimum of 4 peptides contributed to each motif. Underlined peptides were enriched in more than 2 animals (see Table 1) and peptide sequences in bold were synthesised for further screening.

Table S4. Peptides that interact with IgY from chickens infected with *S*. Enteritidis and not *S*. Hadar.

**Motif[a]**	**Contributing peptides [number]**
No motif	**RESP*NSLA, LPRPI*PTP, AP*GPAPTA, PRN*EHFAM, RS*SRRFHL, EP*QSARPS, APHM*FKTS, WARTHV*KV, CSTRRPI*TTC, FVNRALIN*, APSR*HRSW, *GASGRSCR, MALLN*SYP, C*PLQSINITC, QALPKREPP,** CPC*VRRNVGCG, CRA*HFLPPHCG, CVSGRWKMFPCG, ENL*RVNVP, IAT**ASNW, IRLMR*LAA, *APTLWKTA, *AQTNPQTA, *TPLQATTL, RSCK*RHET, SDARFES*V [27]

[a]The MEME algorithm was used to analyse for motifs for the phage-peptide sequences found to be enriched against IgY from multiple chickens infected with *S*. Enteritidis. An E-value cut-off of 0.05 was used and no motifs were found. Peptide sequences in bold were synthesised for further screening.

Are peptides selected to be specific for *S.* Typhimurium infection in pigs also discriminatory for the infection in chickens?

Table S5. ELISA screening of peptide binding to chicken IgY using peptides validated as being discriminatory for *S.* Typhimurium infection in pigs.

Peptide sequence[a]	ELISA assessment	
	AUC of ROC[b]	P value[b]
AEGEFGFTNAIELA	0.68	0.065
AEGEFSQTSLSAAT	0.79	0.003
AEGEFFDARNHPDE	0.83	<0.001
EFCPLEKRISFDVRG	0.83	<0.001
EGEFCPLKTYQL*CAG	0.78	0.003
AEGEF*TTP*SPSQ	0.67	0.080
GEFCDGPNSSSIAAG	0.67	0.080
AEGEFAPQTHSQQP	0.73	0.020
AEGEFLATVASN*M	0.70	0.041
AEGEFVQATDTN*S	0.67	0.086
Control peptide	0.54	0.656

[a]Opal and ochre codons are denoted by an asterisk. Residues coded for by these stop codons were omitted in the synthesised peptides. N-terminal residues AEGEF are phage coat protein residues. Constrained peptides also contain a phage coat protein G residue at their C-terminus.
[d]ROC curves were produced for the recognition of each of 10 peptides with IgY from *S.* Typhimurium infected (n=16) and non-infected (n=20) chickens in an ELISA. AUC and corresponding p values are listed.

Comparison of the efficacy of NGPD using random peptide libraries to other immunoproteomics methods

The presented data demonstrates a method for the identification and validation of peptides that are specifically recognised by polyclonal antibodies upon a particular infection. The method is an alternative approach to more established immunoproteomics methods to identify such disease-specific epitopes/antigens (examples are given in Table S6).

Traditional methods to map polyclonal responses to infection involve the growth of the organism in culture followed by the separation of proteins using 2D SDS-PAGE, probing with polyclonal sera and identification of antigens by mass spectrometry methods or microsequencing. However, these methods have severe limitations imposed by the occurrence of disparate protein expression in culture compared to *in vivo*, the restricted resolution of SDS-PAGE and their suitability only to proteins and

not to other immunogenic macromolecules. In addition, the methods are laborious and expensive and are not high-throughput meaning only limited numbers of sera can be tested during antigen discovery. Such conventional probing of polyclonal antibody responses to infection then usually relies on the production of the identified antigens as recombinant proteins before being assayed in test formats such as ELISAs. The production of recombinant proteins can produce a bottleneck in epitope discovery and can also fail to reproduce conformational epitopes, a type of epitope that usually makes up the majority of the repertoire of B cell epitopes[11,12]. Alternatively, where a particular antigenic protein is investigated, its epitopes can be identified by the empirical testing of overlapping peptides but this technique will usually only identify linear epitopes; alanine scanning can identify both linear and conformational epitopes but is labour intensive and expensive.

Phage display techniques have also been applied to mapping polyclonal antibody responses. These methods use phage display of a large library of ligands that are probed with polyclonal sera. The ligand libraries are usually random peptides and importantly, these random peptides can mimic both linear and conformational protein epitopes as well as epitopes of other macromolecules such as sugars and lipids[13]. Phage-peptide libraries have identified epitopes/mimotopes to infectious disease and vaccinations. However, the methods are not particularly efficient and such conventional phage display panning strategies can often fail to yield any specific binders. This is likely due to the presence of parasitic phage clones (target unrelated peptides) and background phage that are not washed away during phage panning. In addition, when relatively high numbers of panning rounds are performed to enrich for true binders and overcome the presence of background phage then diversity is lost which may limit the number of the true-binders that are identified. For instance, the examples shown in Table S6 detail the screening of hundreds of phage clones that yield proportionally few distinct peptides that were positive in ELISAs.

An alternative phage display approach is the display of the fragmented genome of a pathogen, which can be highly efficient at identifying epitopes/antigens (Table S6). However, this method is labour intensive in terms of the production of a bespoke display library and is also limited to the identification of protein-derived epitopes and in addition does not represent post-translational modifications.

To overcome the limitations of conventional phage display screening, several recent reports use next generation sequencing platforms to sequence sub-libraries from

panning experiments (NGPD). The experiments sequence vastly more peptides than can be screened by conventional phage clone picking following by immunoassay and have identified target-specific ligands even after a single round of panning. The NGPD platforms can identify hundreds to thousands of enriched ligands over background and parasitic phage and the method may have significant application to mapping polyclonal antibody responses to infection. In the present study we demonstrate that NGPD can map polyclonal antibody responses to infections of pigs and chickens with *Salmonella enterica*. The method allows the comparison of peptide enrichment against antibodies derived from different cohorts of animals, for example infected vs non-infected or between animals infected with different related pathogens. The data demonstrates that the identification of peptides that are specific for a particular infection is highly efficient with 60-81% of the peptides identified being validated in ELISAs. In addition, the method does not require bespoke phage library production and peptides should represent a wide range of epitopes (linear, conformational and mimotopes of non-protein structures).

Table S6. Examples of methods for mapping polyclonal antibody recognition of epitopes/antigens of infectious agents.

**Methodology**	**Infectious agent(s)**	**Screening, identification and validation of epitopes/antigens**	
**Western blot analysis followed by matrix-assisted laser desorption/ionization time of flight mass spectrometry**		**Number identified**	**Number validated by immunoassay**
	Coxiella burnetti[11]	20	19
	Brucella abortus[14]	23	Not done
	Bartonella quintana[15]	24	Not done
	Neisseria meningitides[16]	33	20
**Random peptide phage display using immunoassay screening**		**Number of phage isolates screened**	**Number validated by immunoassay**
	Trypanosoma cruzi[17]	198	3
	Mycobacterium leprae[18]	742	3
	Rhipicephalus (Boophilus) microplus using hyperimmune immunised animals[19]	280	16
**cDNA phage display using NGS screening**		**Number of phage isolates screened**	**Number validated by immunoassay**
	Salmonella enterica serovar Typhimurium[20]	184	47
	Mycobacterium tuberculosis[21]	Sequencing revealed 6 antigens	6
**Random peptide phage display using NGS screening**		**Number of phage isolates screened**	**Number validated by immunoassay**
	Salmonella enterica serovars Typhimurium[a]	27	22
	Salmonella enterica serovars Enteritidis[a]	15	9

[a] Data presented in this study

Supplementary references

1. Malik, P., Terry, T.D., Bellintani, F. & Perham, R.N. Factors limiting the display of foreign peptides on the major coat protein of filamentous bacteriophage capsids and a potential role for leader peptidase. *FEBS Letts.* **436**, 263-266 (1998).

2. Merzlyak, A. & Lee, S-W. Engineering phage materials with desired peptide display: ratinal design sustained through natural selection. *Bioconj. Chem.* **20,** 2300-2310 (2009).
3. Felici, F., Castagnoli, L., Musacchio, A., Jappelli, R. & Cesareni, G. Selection of antibody ligands from a large library of oligopeptides expressed on a multivalent exposition vector. *J. Mol. Biol.* **222**, 301-310 **(**1991).
4. Luzzago, A., Felici, F., Tramontano, A., Pessi, A. & Cortese, R. Mimicking of discontinuous epitopes by phage displayed peptides, I. Epitope mapping of human H ferritin using a phage library of constrained peptides. *Gene* **128**, 51-57 (1993).
5. Ryvkin, A., et al. Deep Panning: steps towards probing the IgOme. *PLoS One* **7(8)**, e41469 (2012).
6. Rhodi, D.J., Soares, A.S. & Makowski L. Quantitative assessment of peptide sequence diversity in M13 combinatorial peptide phage display libraries. *J. Mol. Biol.* **322**, 1039-1052 (2002).
7. Derda, R. et al. Diversity of phage displayed libraries of peptides during panning and amplification. *Molecules* **16**, 1776-1803 **(**2011).
8. t Hoen, P.A. et al. Phage display screening without repetitious selection rounds. *Anal. Biochem.* **421**, 622-631 (2012).
9. Matochko, W.L., Cory Li, S., Tang, K.H. & Derda, R. Prospective identification of parasitic sequences in phage display screens. *Nucleic Acids Res.* **42**, 1784-1798 (2014).
10. Bailey, T. & Elkan, C. Fitting a mixture model by expectation maximization to discover motifs in biopolymers, *Proceedings of the Second International Conference on Intelligent Systems for Molecular Biology*, pp. 28-36, AAAI Press, Menlo Park, California (1994).
11. Xiong, X., Wang, X., Wen, B., Graves. S, & Stenos, J. Potential serodiagnostic markers for Q fever identified in *Coxiella burnetii* by immunoproteomic and protein microaraay approaches. *BMC Microbiol.* **12**, 35 (2012).
12. Tjalsma, H., Schaeps, R.M.J. & Swinkels, D. Immunoproteomics: from biomarker discovery to diagnostic applications. *Proteomics Clin. Appl.* **2**,167-180 (2008).
13. Smith, C.M. et al. Peptide mimics of two pneumococcal capsular polysaccharide serotypes (6B and 9V) protect mice from a lethal challenge with *Streptococcus*

pneumoniae. *Eur. J. Immunol.* **39**, 1527-1535 (2009).

14. Lee, J.J. et al. Immunoproteomic identification of immunodominant antigens independent of the time of infection in *Brucella abortus* 3308-challenged cattle. *Vet. Res.* **46**, 17 (2015).
15. Boonjakuakul, J.K. et al. Proteomic and immunoblot analysis of *Bartonella Quintana* total membrane proteins identify antigens recognized by sera from infected patients. *Inf. Immun.* **75**, 2548-2561 (2007).
16. Mendum, T.A., Newcombe, J., McNeilly, C. & McFadden J. Towards the immunoproteome of *Neisseria meningitides*. *PloS ONE* **4(6)**: e5940.
17. Toledo-Machado, C.M. et al. Immunodiagnosis of canine visceral leishmaniasis using mimotope peptide selection from phage displayed combinatorial libraries. *BioMed Res. Intl.* **2015,** Article ID 401509 (2015)
18. Alban, S.M., de Moura J.F., Minozzo, J.C., Mira M.T. & Soccol, V.T. Identification of mimotopes of *Mycobacterium leprae* as potential diagnostic reagents. *BMC Infect. Dis.* **13**, 42 (2013).
19. Prudencio, C.R., Rodrigues, A.A.R., Cardoso, R., Szabó, M.P.J. & Goulart, L.R. Antigen fingerprinting of polyclonal antibodies in immunized chickens with tick total proteins: a reservoir for the discovery of novel antigens. *J. Biomol. Screen* **16,** 1027-1036 (2011).
20. Meyer, T. et al. Identification of immunogenic proteins and generation of antibodies against *Salmonella* Typhimurium using phage display. *BMC Biotechnol* **12**, 29 (2012).
21. Liu, S. et al. Subtractive screening with the Mycobacterium tuberculosis surface protein phage display library. *Tuberculosis* **91**, 579-586 (2011).
